# Comparison of distraction arthroplasty alone versus combined with arthroscopic microfracture in treatment of post-traumatic ankle arthritis

**DOI:** 10.1186/s13018-017-0546-7

**Published:** 2017-03-17

**Authors:** Kaibin Zhang, Yiqiu Jiang, Jing Du, Tianqi Tao, Wang Li, Yang Li, Jianchao Gui

**Affiliations:** Department of Orthopaedics, Nanjing First Hospital, Nanjing Medical University, ChangLe Road 68, Nanjing, 210000 Jiangsu Province People’s Republic of China

**Keywords:** Distraction arthroplasty, Microfracture, Ankle arthritis, Arthroscopy, Cartilage

## Abstract

**Background:**

This study aims to compare clinical outcomes of distraction arthroplasty alone versus combined with arthroscopic microfracture in treating post-traumatic ankle arthritis.

**Methods:**

The study cohort consisted of 96 patients (96 ankles) who underwent distraction arthroplasty alone or combined with arthroscopic microfracture between May 2005 and April 2012. Patients were divided into the distraction group (*n* = 46) and the combined group (*n* = 50). The American Orthopaedic Foot and Ankle Society (AOFAS) score, visual analog scale (VAS), and ankle activity score (AAS) were used to compare the clinical outcomes between groups. Arthritis severity was assessed on the radiograph.

**Results:**

At the mean follow-up period of 30.8 ± 3.1 and 31.4 ± 3.6 months, respectively, no severe complications occurred and no further surgical interventions for symptomatic arthritis were required in both groups. The AOFAS scores improved significantly in the combined group than in the distraction group (59.0 ± 4.7 and 58.0 ± 4.9 preoperatively versus 85.0 ± 4.9 and 88.9 ± 5.4 at final visit, *P* < 0.001). The AAS scores were also significantly higher in the combined group (3.6 ± 1.1 and 3.3 ± 1.0 preoperatively versus 6.5 ± 1.1 and 7.1 ± 1.3 at final visit, *P =* 0.009). Pain was significantly alleviated in the combined group by the VAS scores (6.4 ± 0.9 and 6.7 ± 0.9 preoperatively versus 2.3 ± 0.8 and 2.0 ± 0.7 at final visit, *P =* 0.040). The combined group achieved better radiographic arthritis severity decrease than the distraction group (*P =* 0.012).

**Conclusions:**

Compared to distraction arthroplasty alone, distraction arthroplasty combined with arthroscopic microfracture can offer better functional recovery, pain relief, and ankle arthritis resolution for treating post-traumatic ankle arthritis.

## Background

Post-traumatic arthritis is a clinically very common disease accounting for 65–80% of secondary ankle arthritis, which can cause severe pain and limited joint mobility. A large number of persons worldwide are affected by this disease [1, 2]. Generally, post-traumatic arthritis is characterized by articular cartilage damage to varying degrees [3]. The goals for treatment of ankle arthritis include pain relief, improvement of joint function, and prevention of further osteoarthritic progression. Various surgical techniques have been used to treat post-traumatic arthritis, such as ankle arthrodesis, total ankle arthroplasty, and distraction arthroplasty [4].

Bone marrow stimulation procedures, such as microfracture and multiple drilling, have the ability of recruiting potential mesenchymal stem cells to repair damaged articular cartilage, which is helpful for treatment of ankle arthritis [5].

Nakasa et al. [6] reported the effectiveness of distraction arthroplasty with arthroscopic microfracture on rheumatoid ankle arthritis, with arthritic joint symptoms significantly improved. Besides, many previous studies demonstrated good-to-excellent clinical results of distraction arthroplasty on ankle arthritis, after which patients achieved good functional outcomes and pain relief [7, 8]. But to date, no studies have reported outcome comparison between distraction arthroplasty with microfracture and distraction arthroplasty alone on post-traumatic arthritis.

The purpose of the present study was to compare clinical outcomes of distraction arthroplasty alone or in combination with arthroscopic microfracture in treating post-traumatic ankle arthritis. We hypothesized that combination of distraction arthroplasty and arthroscopic microfracture surgery would result in function improvement, pain relief, and arthritis severity decrease when compared to distraction arthroplasty surgery alone.

## Methods

The Institutional Review Board of Nanjing First Hospital approved this study (Permit Number 20051036). Detailed information about the surgical interventions was provided to all patients. A written informed consent was obtained from each patient. We also obtained consent to publication of their medical data, including medical records, photographs, and images. All procedures were performed in accordance with the World Medical Association’s Declaration of Helsinki.

### Patients

A total of 119 patients (123 ankles) with post-traumatic ankle arthritis underwent distraction arthroplasty alone or combined with arthroscopic microfracture from May 2005 to April 2012 at the Department of Orthopaedics, Nanjing First Hospital, Nanjing Medical University (Nanjing, China). They had to have a minimum 2-year follow-up after treatment.

Inclusion criteria were as follows: symptomatic post-traumatic ankle arthritis with unilateral ankle affected, age between 18 and 60 years, absent of ankle joint infection or significant periarticular deformity, primary surgery, and failed non-operative treatment. Twenty patients (24 ankles) were excluded from final analysis due to the reasons listed below: bilateral lesions (*n =* 4), an age younger than 18 years or older than 60 years (*n =* 12), ankle joint infection (*n =* 3), and major periarticular deformity (*n =* 1). Another three patients (3 ankles) were also excluded because of the follow-up less than 24 months. The remaining 96 patients (96 ankles) constituted the study cohort. They were divided into groups of treatment of distraction arthroplasty alone (distraction group; *n =* 46) and distraction arthroplasty combined with arthroscopic microfracture (combined group; *n =* 50).

### Surgical technique

All operations were performed under general anesthesia by a senior surgeon (Jianchao Gui). Two grams of cefazolin sodium hydrate as a prophylactic antibiotic was given intravenously before surgery. The patients were operated on using a tourniquet in the supine position.

**Fig. 1:**
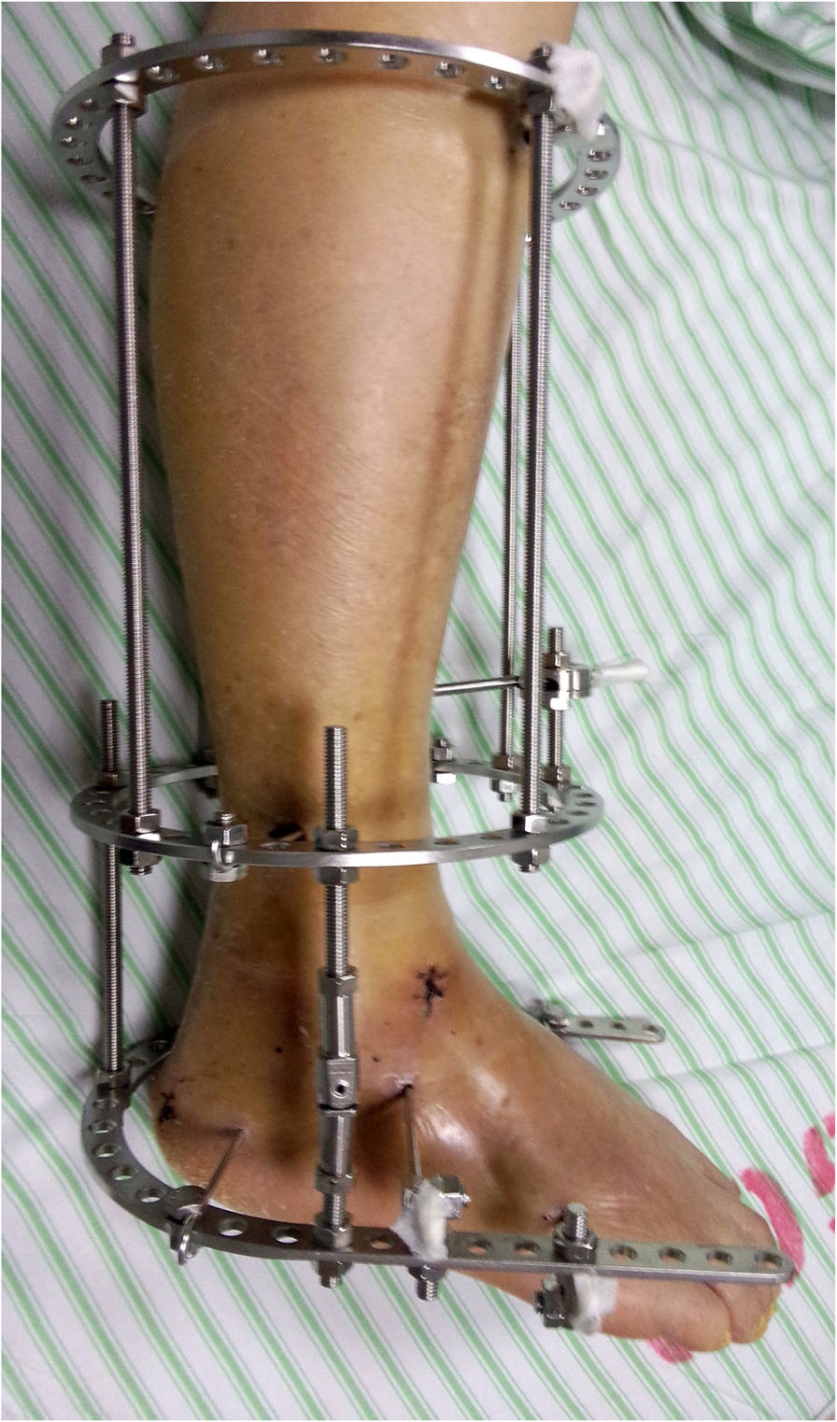
Application of the Ilizarov fixator for distraction arthroplasty. The overall appearance demonstrated successful application of the Ilizarov fixator. Distraction with motion was performed by use of distraction rods with hinges. Patients were allowed to walk with partial weight-bearing for at least 3 months, followed by the Ilizarov fixator being removed

For patients undergoing distraction arthroplasty alone, two Kirschner wires were drilled at different angles proximally and distally through the tibia and fixed under tension (1.3 kN) to an external ring. They were connected by screw-threaded rods. Two pins were drilled through the calcaneus. They were fixed to a half ring around the heel. Another two pins were then drilled through the metatarsals and tensioned to a half ring over the forefoot. Both half rings were connected to each other by angle plates and straps by which the foot was stabilized. Finally, the fixation of the tibia and the foot was connected by Ilizarov lengthening rods which allowed controlled distraction of the talus and tibia. The overall appearance of successful application of the Ilizarov fixator is seen in Fig. 1.

**Fig. 2:**
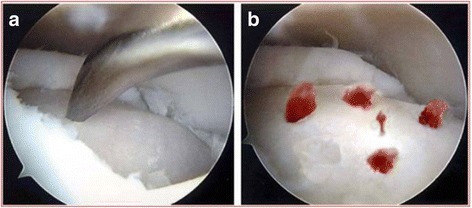
The arthroscopic photograph showing procedures of arthroscopic microfracture. **a** Microfracture was performed on the talar dome. **b** Blood outflowed from the microfracture holes after tourniquet release

For patients undergoing distraction arthroplasty combined with microfracture procedures, standard anteromedial and anterolateral portals were used with non-invasive distraction by an ankle distractor firstly (Smith & Nephew, Memphis, TN). A 2.7-mm, 30° oblique arthroscope was used. Synovectomy was performed firstly, followed by removal of the osteophytes and curettage of any unstable cartilage fragments. The arthroscopic microfracture procedure was performed on the talar dome. Each microfracture was created 3 to 4 mm apart to a depth of approximately 3 mm (Fig. 2). After the fat droplet and blood outflowed from the microfracture holes, the Ilizarov fixator was applied according to the same procedures as the distraction group.

**Fig. 3:**
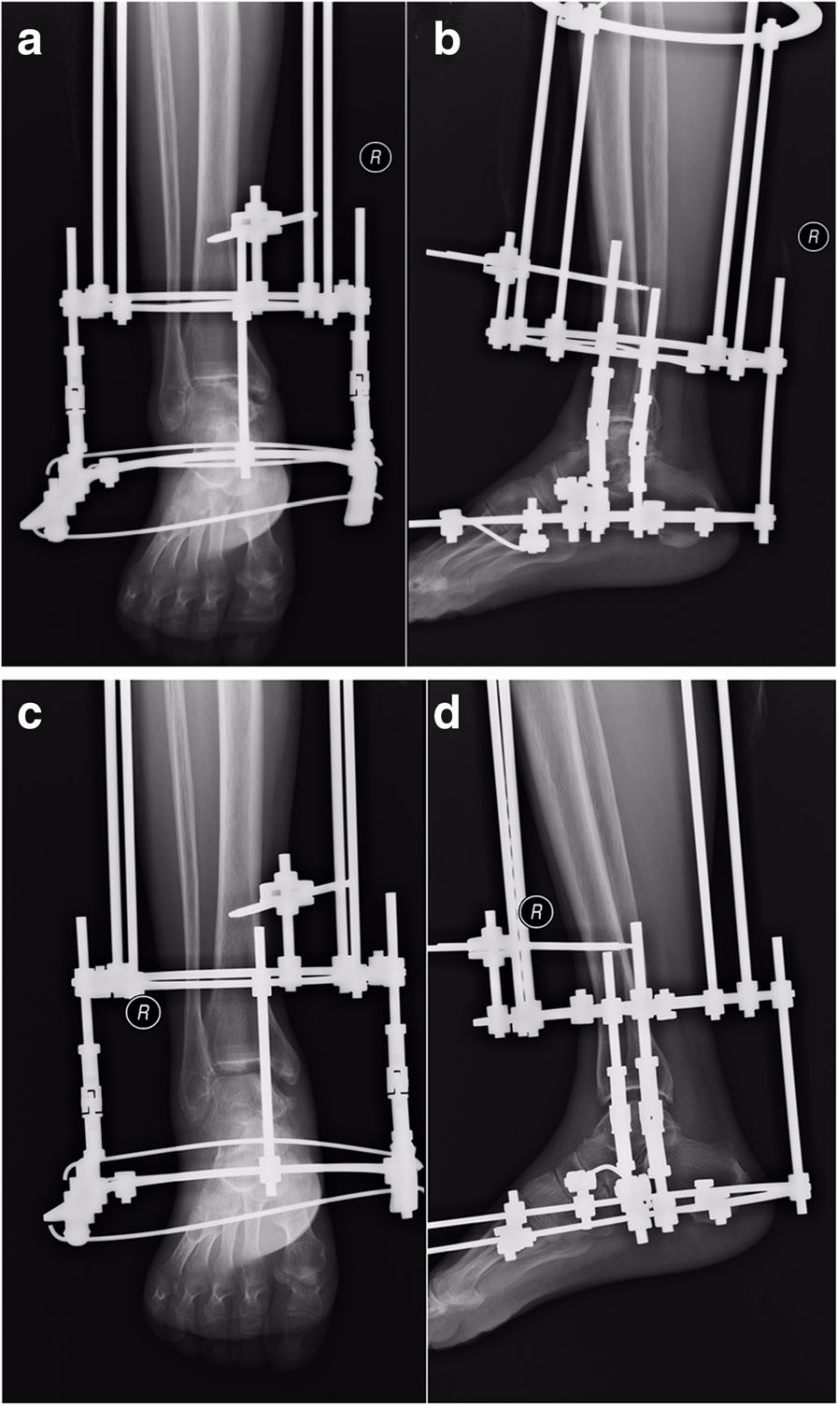
The radiographic views of the ankle before and after distraction. **a**, **b** The anteroposterior and lateral radiographs taken 1 day postoperatively showed the Ilizarov fixator was fixed appropriately. **c**, **d** The anteroposterior and lateral radiographs taken 1 week postoperatively showed joint space significantly enlarged due to 0.5-mm-distraction arthroplasty twice daily for five consecutive days

Postoperatively, 4 g of cefazolin sodium hydrate was given within 48 h. For the patients in both groups, distraction was carried out with a distance of 5 mm (0.5 mm twice daily for five consecutive days) from the day after surgery (Fig. 3). The patients were allowed to walk bearing weight gradually 7 days postoperatively. Three months after initiation of treatment, the Ilizarov fixator was removed for both groups.

### Clinical evaluation

The operative time of every surgery was obtained according to operation notes from patients’ medical records. The clinical evaluations consisted of functional evaluations and pain assessment. Functional evaluations included the American Orthopaedic Foot and Ankle Society (AOFAS) ankle-hindfoot score as well as the ankle activity score (AAS) developing from the Tegner scoring system [9]. The 100-point AOFAS scoring system combines subjective and objective criteria to evaluate clinical parameters; points are allocated as follows: pain = 40, function = 50, and alignment = 10. This system considers a score of ≥90 as excellent, 80–89 as good, 70–79 as fair, and ≤69 as poor. Pain was evaluated by the 10-point visual analog scale (VAS). To avoid examiner bias, clinical evaluations were performed by two independent observers (Kaibin Zhang and Jing Du) who were not involved in the surgical treatment of the patients.

### Radiographic examination

Standard radiographs (anteroposterior and lateral radiographs) were obtained before surgery and at the final follow-up. An arthrosis evaluation was performed according to the Takakura Radiologic Arthrosis Classification System [10] on the radiograph by two independent observers (Yiqiu Jiang and Tianqi Tao) who were not involved in the surgical treatment of the patients.

### Statistical analysis

All the continuous data were expressed as mean ± standard deviation (SD). Normality was tested using the Kolmogorov–Smirnov test. Intra-group and intergroup comparisons of normally distributed continuous variables were performed using paired *t* test and independent *t* test, respectively. Wilcoxon rank test and Mann–Whitney *U* test were used for intra- and intergroup comparisons of continuous variables without normal distribution, respectively. A Pearson chi-square test was used to compare categorical variables between groups. All data were analyzed using the SPSS 19.0 statistical software (SPSS Inc., Chicago, IL, USA). Statistical significance was accepted for *P* values less than 0.05.

## Results

### Baseline characteristics

The distraction group consisted of 18 men and 26 women with a mean age of 43.6 ± 9.3 years (range, 23–59 years) and mean follow-up duration of 30.8 ± 3.1 months (range, 24–39 months). The combined group consisted of 20 men and 30 women with a mean age of 41.8 ± 8.7 years (range, 20–58 years) and a mean follow-up duration of 31.4 ± 3.6 months (range, 24–40 months).

The baseline characteristics of the patients in both groups are summarized in Table 1. In terms of causes of post-traumatic ankle arthritis, the distraction group was ankle fractures in 22 cases (47.8%), recurrent sprains in 10 cases (21.7%), persistent ankle instability in 8 cases (17.4%), ankle dislocations in 5 cases (10.9%), and other in 1 case (2.2%). And the combined group was ankle fractures in 24 cases (48.0%), recurrent sprains in 9 cases (18.0%), persistent ankle instability in 10 cases (20.0%), ankle dislocations in 6 cases (12.0%), and other in 1 case (2.0%). The preoperative baseline data including age, gender, affected side, body mass index (BMI), duration of symptoms, and duration of follow-up did not show significant differences between the two groups (*P* > 0.05 for all parameters).

**Table 1:** Comparison of the baseline data of the patients between the distraction group and the combined group

	Distraction group (*n =* 46)	Combined group (*n =* 50)	*P* value*
Age (years)	43.6 ± 9.3	41.8 ± 8.7	n.s.
Gender (male/female)	18/26	20/30	n.s.
Body mass index (BMI, kg/m^2^)	24.6 ± 3.4	25.1 ± 3.8	n.s.
Side (left/right)	20/26	22/28	n.s.
Duration of symptoms (months)	21.2 ± 6.7	20.4 ± 6.8	n.s.
Follow-up duration (months)	30.8 ± 3.1	31.4 ± 3.6	n.s.
Causes (%)			n.s.
Ankle fractures	22 (47.8%)	24 (48.0%)	
Recurrent sprains	10 (21.7%)	9 (18.0%)	
Persistent ankle instability	8 (17.4%)	10 (20.0%)	
Ankle dislocations	5 (10.9%)	6 (12.0%)	
Other	1 (2.2%)	1 (2.0%)	

Unless otherwise stated, the data are presented as mean ± SD

*Independent *t* test or chi-square test. The *P* values shown are for intergroup comparisons. Significance was accepted for *P* values of <0.05

### Clinical outcomes

The clinical outcomes between the two groups are listed in Table 2. For the patients in both groups, no severe perioperative complications occurred, for example, nerve injury, deep infection, and deep vein thrombosis. And none of the patients presented symptomatic arthritis that required further surgical intervention during the follow-up period. Three months after surgery, the Ilizarov frame was removed. No ankle joint stiffness, recurrence of ankle fracture, and ankle joint swelling were observed in both groups.

**Table 2:** Comparison of clinical outcomes between the distraction group and the combined group

Outcomes	Distraction group (*n =* 46)	Combined group (*n =* 50)	*P* value*
Operative time (min)	47.4 ± 7.1	60.6 ± 12.1	<0.001
AOFAS			
Preoperative	59.0 ± 4.7	58.0 ± 4.9	n.s.
Final follow-up	85.0 ± 4.9	88.9 ± 5.4	<0.001
AAS			
Preoperative	3.6 ± 1.1	3.3 ± 1.0	n.s.
Final follow-up	6.5 ± 1.1	7.1 ± 1.3	0.009
VAS			
Preoperative	6.4 ± 0.9	6.7 ± 0.9	n.s.
Final follow-up	2.3 ± 0.8	2.0 ± 0.7	0.04

Unless otherwise stated, the data are presented as mean ± SD

*AAS* ankle activity score, *AOFAS* American Orthopaedic Foot and Ankle Society ankle-hindfoot score, *VAS* visual analog scale

*Independent *t* test. The *P* values shown are for intergroup comparisons. Significance was accepted for *P* values of <0.05

Compared with the distraction group, the mean operative time of the combined group was longer (47.4 ± 7.1 and 60.6 ± 12.1 min, respectively, *P <* 0.001). The mean AOFAS score was 59.0 ± 4.7 (range, 50–68) in the distraction group and 58.0 ± 4.9 (range, 52–69) in the combined group preoperatively. These significantly improved to 85.0 ± 4.9 (range, 77–95) and 88.9 ± 5.4 (range, 77–97) at the final follow-up, respectively (*P <* 0.05, for both groups). The mean AAS score was 3.6 ± 1.1 (range, 2–6) in the distraction group and 3.3 ± 1.0 (range, 2–5) in the combined group preoperatively. These significantly increased to 6.5 ± 1.1 (range, 5–9) and 7.1 ± 1.3 (range, 5–9) at the final follow-up, respectively (*P <* 0.05, for both groups). The mean VAS score was 6.4 ± 0.9 (range, 5–9) in the distraction group and 6.7 ± 0.9 (range, 5–8) in the combined group preoperatively. These also significantly decreased to 2.3 ± 0.8 (range, 0–4) and 2.0 ± 0.7 (range, 0–4) at the final follow-up, respectively (*P <* 0.05, for both groups). There were no significant differences between the preoperative AOFAS, VAS, and AAS scores in both groups (*P* > 0.05). But at the final follow-up, the AOFAS and AAS scores of the combined group were higher than those of the distraction group (*P <* 0.001 and *P =* 0.009), with the VAS score lower than that of the distraction group (*P =* 0.040).

According to the AOFAS scores, the overall results were excellent in 23 cases (50.0%), good in 18 (39.1%), and fair in 5 (10.9%) in the distraction group, with the excellent and good rate 89.1%. In the combined group, they were excellent in 28 cases (56.0%), good in 19 (38.0%), and fair in 3 (6.0%), with the excellent and good rate 94.0%.

### Radiological outcomes

**Fig. 4:**
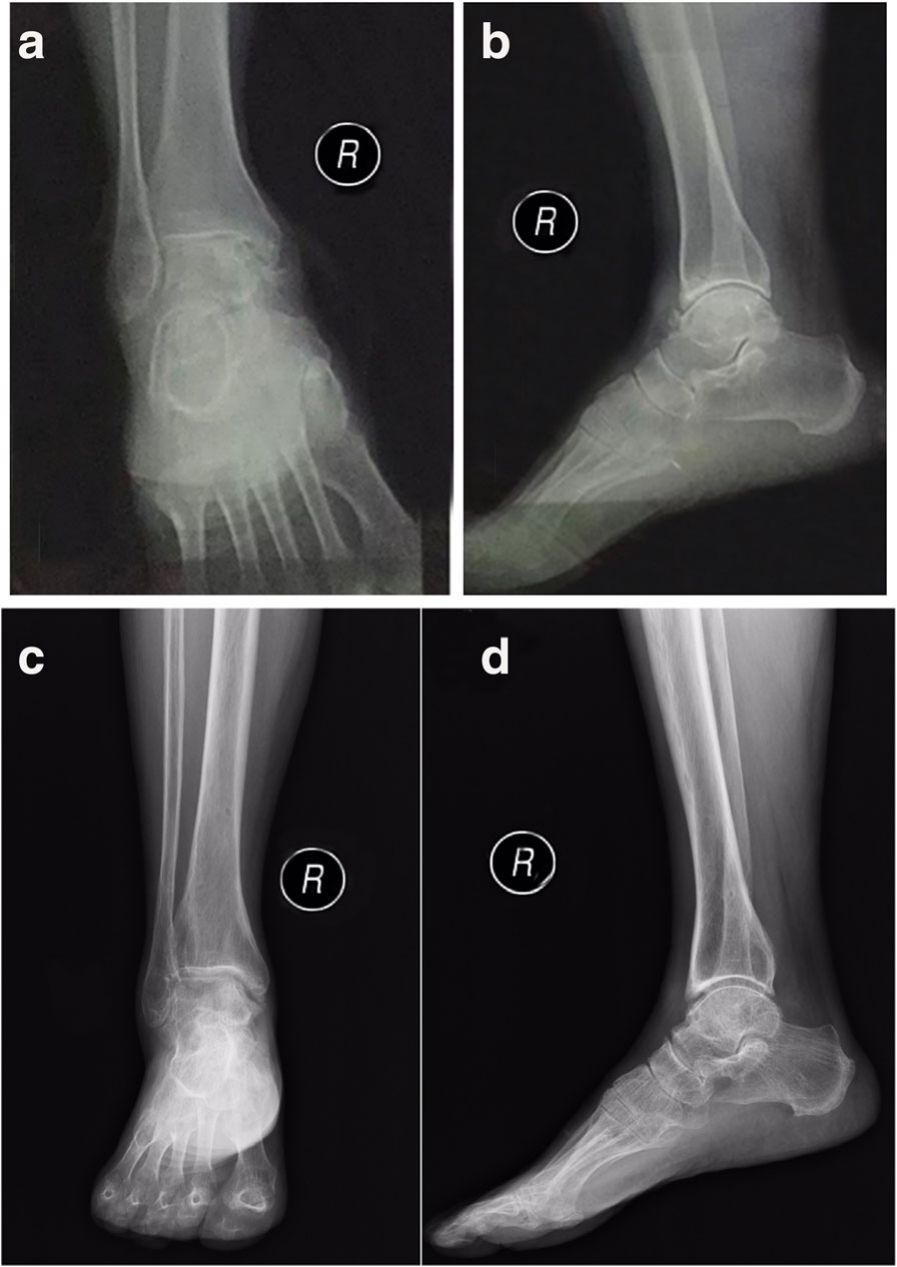
The typical radiographic views of the ankle before and after distraction arthroplasty combined with arthroscopic microfracture. **a**, **b** Preoperative anteroposterior and lateral radiographic views of the ankle suggested post-traumatic ankle arthritis characterized by osteophyte formation and obliteration of the joint space. **c**, **d** At the final follow-up of 28 months postoperatively, anteroposterior and lateral radiographic views of the ankle demonstrated joint-space enlargement and absence of osteophyte

The radiological outcomes of both groups are summarized in Table 3. Most of the patients had radiological resolution of traumatic arthritis after the treatment. The typical radiographs of the ankle before and after the combined surgery are shown in Fig. 4.

**Table 3:** Preoperative and postoperative radiographic stages in the distraction group and the combined group

	Distraction group (*n =* 46)		Combined group (*n =* 50)		
Stage^a^	Preoperative	Postoperative	Preoperative	Postoperative	*P* value*
0 (normal)	–	14 (30.4%)	–	20 (40.0%)	0.012
I	8 (17.4%)	16 (34.8%)	10 (20.0%)	19 (38.0%)	
II	18 (39.1%)	15 (32.6%)	24 (48.0%)	11 (22.0%)	
III	20 (43.5%)	1 (2.2%)	16 (32.0%)	–	
IV	–	–	–	–	

Data are reported as *n* (%)

*Chi-square test. The *P* values shown are for intergroup comparisons. Significance was accepted for *P* values of <0.05

^a^Determined by Takakura Radiologic Arthrosis Classification System

The results of the preoperative staging system using plain radiographs were as follows: stage I, 8 ankles (17.4%); II, 18 ankles (39.1%); and III, 20 ankles (43.5%) in the distraction group; and stage I, 10 ankles (20%); II, 24 ankles (48%); and III, 16 ankles (32%) in the combined group. At the final follow-up, the distraction group included 14 ankles (30.4%) at stage 0, 16 ankles (34.8%) at stage I, 15 ankles (32.6%) at stage II, and 1 ankle (2.2%) at stage III. The combined group included 20 ankles (40.0%) at stage 0, 19 ankles (38.0%) at stage I, and 11 ankles (22.0%) at stage II.

In terms of stage improvements, there were no improvement in 6 ankles (13.0%), one grade improvement in 17 ankles (37.0%), two grades in 19 ankles (41.3%), and three grades in 4 ankles (8.7%) in the distraction group. In contrast, there were no improvement in 5 ankles (10.0%), one grade improvement in 18 ankles (36.0%), two grades in 20 ankles (40.0%), and three grades in 7 ankles (14.0%) in the combined group. There were no significant differences in preoperative radiological arthrosis grades between the two groups, but at the final follow-up, the radiological arthrosis grades were significantly decreased in the combined group compared to those in the distraction group (*P =* 0.012).

## Discussion

To the best of our knowledge, this was the first retrospective study that compared the outcomes of distraction arthroplasty combined with microfracture versus distraction arthroplasty alone in treatment of post-traumatic arthritis. The most important finding of the present study was that compared to distraction arthroplasty alone, distraction arthroplasty with arthroscopic microfracture would result in improved postoperative functional recovery, pain relief, and arthritis resolution, as determined by AOFAS, VAS, and AAS score as well as radiographic ankle arthritis grades. In terms of the AOFAS score, the combined surgery achieved an excellent and good rate of 94.0%, which was higher than that of the distraction arthroplasty alone (89.1%). Although the operative time of combined surgery was longer than that of distraction alone (60.6 ± 12.1 versus 47.4 ± 7.1 min, respectively), we thought it worthy of spending time for achieving better clinical outcomes.

Many previous studies have reported good outcomes of distraction arthroplasty in ankle rheumatoid arthritis [6], ankle osteoarthritis [11], and post-traumatic ankle arthritis [12]. Van Valburg et al. [13] applied the Ilizarov apparatus across the ankle joint with a 5-mm distraction in patients of post-traumatic arthritis. Their results showed most of patients experienced range of movement improvement and pain relief, which was consistent with our findings. The mechanism underlying the distraction arthroplasty is likely associated with cartilage regeneration and intermittent flow of intra-articular synovial fluid [14]. Cartilage injury is common in post-traumatic ankle joint. The mechanical stress on the joint surface is thought to cause further wear and tear and therefore inhibit articular cartilage-healing process. Treatment by distraction arthroplasty can unload mechanical stress in the absence of axial loading, which would benefit regeneration of the cartilage [15, 16].

Arthroscopic microfracture is widely used by many surgeons in treating osteochondral lesions of the talus. Polat et al. [17] and Park and Lee [18] reported excellent results of arthroscopic microfracture in the treatment of talus osteochondral lesions. It is thought that bone marrow stimulation procedures, such as microfracture, are based on the principle that they can promote blood flow in the debrided cartilage lesion, allowing for an influx of potential mesenchymal stem cells from the bone marrow. This process can initiate formation of fibrous cartilage, which therefore may exert a synergistic effect along with distraction arthroplasty benefiting for repair of damaged articular cartilage [19, 20]. Accordingly, in the present study, we combined distraction arthroplasty and arthroscopic microfracture technique, which demonstrated excellent functional and clinical outcomes on treating post-traumatic ankle arthritis.

Ankle arthroscopy is a minimally invasive surgical method with few complications and low postoperative morbidity. In the present study, no patient presented major complications, such as deep infection or deep vein thrombosis. Arthroscopic microfracture also has an advantage of avoiding potential heat necrosis, which is common in another bone marrow stimulation procedure arthroscopic drilling [21]. However, it is noted that there is a disadvantage of microfracture in that regenerated articular cartilage is fibrous cartilage instead of the original hyaline cartilage. The fibrous cartilage is softer than hyaline cartilage and is easily damaged due to low biomechanical strength [22]. Another potential risk is that microfracture may create loose bodies, which may cause locking and cartilage damage if not properly removed [23, 24]. So at the end of the arthroscopy surgery, we all carefully inspected the ankle joint for removing visible loose bodies. And we did not detect any patient with locking or loose bodies on plain radiographs.

Our study also has several limitations. Firstly, the present study is a single-center retrospective study with relatively small sample size, and our follow-up period is relatively short. Secondly, we mainly assessed clinical outcomes in aspects of function and pain, not including follow-up MRI and second-look arthroscopy. So a prospective multi-center controlled study involving more cases with long-term follow-up is required in the future.

## Conclusions

In conclusion, when compared to distraction arthroplasty alone, distraction arthroplasty combined with arthroscopic microfracture can offer better functional recovery, pain relief, and significant radiologically ankle arthritis severity decrease for patients with post-traumatic ankle arthritis. And no severe complications occurred and no further surgical interventions for symptomatic arthritis were required during the follow-up period of combined surgery. Distraction arthroplasty combined with arthroscopic microfracture is a good option for post-traumatic ankle arthritis, on which orthopedic surgeons should put more emphasis in the future.

## Abbreviations

AAS: Ankle activity score
AOFAS: American Orthopaedic Foot and Ankle Society
MRI: Magnetic resonance images
VAS: Visual analog scale
